# Investigating comparative polymerase chain reaction for antigen receptor rearrangement analysis in different types of feline lymphoma samples

**DOI:** 10.3389/fvets.2024.1439068

**Published:** 2024-08-30

**Authors:** Jedsada Siripoonsub, Somporn Techangamsuwan, Sirintra Sirivisoot, Araya Radtanakatikanon, Anudep Rungsipipat

**Affiliations:** Center of Excellence for Companion Animal Cancer, Department of Pathology, Faculty of Veterinary Science, Chulalongkorn University, Bangkok, Thailand

**Keywords:** feline lymphoma, clonality, T-cell, B-cell, PARR (PCR to detect antigen receptor rearrangements)

## Abstract

Cats have the highest incidence of lymphoma among all animal species. Lymphoma accounts for 41% of all malignant tumors in cats and is responsible for 90% of hematopoietic tumors in felines. Biopsies are considered the gold standard for diagnosis. Polymerase chain reaction (PCR)-based clonality assessment of antigen receptor gene rearrangements can be a valuable complementary tool for identifying infiltrating B-and T-lymphocyte clones. Many studies have focused on intestinal cases but few have addressed mediastinal lymphoma. This study aims to: (1) investigate the clonality patterns of lymphoma samples from various anatomical sites, with a particular focus on mediastinal lymphoma, and (2) evaluate the sensitivity and specificity of the clonality analysis of pleural effusion samples in comparison with cytology, histology, immunohistochemistry, and immunocytochemistry for diagnosing mediastinal lymphoma. There were 82 cases, divided into 49 formalin-fixed and paraffin-embedded biopsy specimens (FFPE), 22 cell pellets, and 11 fresh tissue. This study examined the sensitivity and specificity of PCR for antigen receptor rearrangement (PARR) compared to immunohistochemistry (IHC) and immunocytochemistry. For T-cell receptor gamma chain genes, PARR demonstrated a sensitivity of 58.33% for both fresh tissue and FFPE samples, with a specificity of 100%. Cell pellet analysis exhibited a sensitivity of 64.71% and maintained 100% specificity. A combined analysis of fresh tissue and FFPE with cell pellets showed a sensitivity of 62.07%. For IGH, the sensitivity for fresh tissue and FFPE samples was 56.25%, while cell pellet analysis showed a sensitivity of 62.50%. When considering fresh tissue and FFPE samples, the sensitivity was 57.14%. In conclusion, molecular techniques have emerged as valuable tools for detecting lymphoma, especially in cases where traditional diagnostic methods yield inconclusive results, such as mediastinal lymphoma. While biopsy may not always be feasible, cytology and cell pellets obtained from pleural effusion offer alternative immunocytochemistry and molecular analysis samples, provided they are of sufficient quality and quantity. All sample types considered in this study were suitable for PARR to aid in cases with inconclusive results. Therefore, the sample selection should be tailored to the clinical situation.

## Introduction

Among all species, cats exhibit the highest prevalence of lymphoma. This type of cancer constitutes 41% of all malignant tumors observed in felines and 90% of hematopoietic tumors observed in cats (1). Feline lymphoma can originate in a chronic inflammatory environment in which neoplastic lymphocytes are mixed with reactive lymphocytes, adding complexity to diagnoses (2).

Feline lymphoma can be classified into alimentary, mediastinal, multicentric, and extranodal according to the anatomic system (3). Of these, the most common is alimentary lymphoma, followed by extranodal, mediastinal, and multicentric lymphoma (4, 5). The World Health Organization (WHO) released the Revised European–American Lymphoma classification for domestic animals adapted from human medicine. This classification was expanded to include other hematopoietic myeloid tumors with the input of a panel of veterinary experts appointed by the WHO (6).

Lymphoma diagnosis involves techniques such as fine-needle aspiration (FNA) cytology, histopathological examination, immunophenotyping, flow cytometry, and molecular diagnosis. Few studies have demonstrated that flow cytometry helps confirm the diagnosis of feline lymphoma (7, 8). A study has shown that flow cytometry alone does not help confirm the diagnostic suspicion of mediastinal lymphoma (9). Histology from tissue biopsies is considered the gold standard method for diagnosing feline lymphomas (10). However, this invasive method might not always provide clear answers due to challenges in assessing the quantity and quality of the lymphocytic infiltrate. When distinguishing between reactive and malignant criteria proves difficult with cytology or histopathology alone, additional assessments are necessary. The polymerase chain reaction (PCR)-based clonality assessment of antigen receptor gene rearrangements can be a valuable complementary tool for identifying infiltrating B-and T-lymphocyte clones (10, 11). While clonality testing helps monitor neoplastic clones, it is crucial to supplement this evaluation with morphology, immunohistochemistry (IHC), and clinical indicators (11).

Clonality assay technology has been introduced as a complementary tool for identifying a clonal population of lymphocytes and determining whether they originate from B-cells or T-cells (5). Molecular clonality analysis is used to diagnose atypical, mixed, or mature lymphoid proliferations, even with minimal sample quantities or in cases where the tissue structure is absent (11). The rearrangement of the immunoglobulin heavy chain (IGH) variable region and T-cell receptor gamma chain (TCRG) genes occurs early in lymphoid differentiation through random recombination of variable (V), diverse (D), and joining (J) regions (10). Gene rearrangement is unique for every lymphocyte clone. TCRG rearrangement occurs in most T-cells, regardless of the surface TCRG phenotype (12). In B-cells, diversity within the complementary determining region 3 (CDR3) of the IGH V gene was assessed using PCR (13), where TCRG primer sets showed an overall sensitivity of 91% and a specificity of 90%. For B-cells, the overall sensitivity was 44% and specificity was 90% (10). Due to its constrained germline repertoire, the TCRG locus is a prominent target for T-cell clonality testing. This restitution minimizes the necessity for a large number of PCR primers. Moreover, TCRG gene rearrangement occurs in αβ and γδ T-cells, enhancing its utility in clonality assessments (14). However, detecting B-cell neoplasms poses a challenge due to somatic hypermutation in the IGH receptor, which can alter nucleotides at primer sites. This alteration can compromise primer binding and potentially result in false-negative results (1, 2).

The sensitivity for detecting a clonal TCRG by PCR in T-cell neoplasms is relatively high, typically reaching values of 79% or higher (5, 10, 15). In contrast, the sensitivity for detecting a clonal IGH receptor among feline B-cell neoplasms has historically been lower, with values ranging from 34 to 89% (1, 10, 16). The identification of clonality is a crucial feature of neoplastic cells. Detecting a clonal population of lymphocytes in tissue specimens can be instrumental in diagnosing lymphoid neoplasia (1). In veterinary medicine, it is strongly recommended that PCR-derived patterns be interpreted according to the EuroClonality/BIOMED-2 guidelines for clonality testing, similar to those used in humans (2, 10, 11, 17).

Samples from lymph nodes, such as the submandibular, popliteal, or axillary, are relatively easy to collect. However, obtaining samples from locations such as the mediastinal lymph nodes for histopathology can be challenging (9). Cytology may sometimes be inconclusive due to poor sample quality. Despite the challenges in sample collection, cytology remains a valuable diagnostic tool for these cases (18). Several authors have deemed DNA extracted from cytology samples suitable for various molecular tests. The sensitivity and specificity of TCRG and IGH V gene rearrangements have been extensively studied, with most studies focusing on alimentary lymphoma cases (5, 19). However, only a few studies have included this anatomical type of sampling for mediastinal lymphoma (20, 21).

This study aims to: (1) investigate the clonality patterns of lymphoma samples from various anatomical sites, with a particular focus on mediastinal lymphoma, and (2) evaluate the sensitivity and specificity of the clonality analysis of pleural effusion samples in comparison with cytology, histology, immunohistochemistry, and immunocytochemistry for diagnosing mediastinal lymphoma.

## Materials and methods

### Study design

Cases were routinely submitted for pathological examination at the Department of Pathology, Faculty of Veterinary Science, Chulalongkorn University, from 2011 to 2023. Diagnosis via cytology, fluid analysis, and histology consistently revealed either lymphoma or reactive lymph node conditions. IHC were employed, using CD3 (IR503, DAKO, Denmark) for T-cell identification and CD20 (AB27093, Abcam, MA, United States), CD79α (SC-53208, SCBT, Dallas, United States), PAX5 (GA650, Dako, CA, United States) for B-cell identification (data not published). Immunocytochemistry (ICC) uses the same antibodies as IHC but specifically employs CD20 and CD79a for identifying B-cells. For comprehensive molecular characterization, PCR for antigen receptor rearrangements (PARR) was conducted on feline B-cell neoplasms, T-cell neoplasms, and negative control samples.

### Case selection

Samples were collected from cats showing signs of lymphadenopathy that strongly suggested lymphoma, including both fresh tissue and formalin-fixed paraffin-embedded (FFPE) specimens. Additionally, cell pellet samples were obtained from the pleural effusion of cats with mediastinal lymphoma. Imaging techniques, such as thoracic radiography and ultrasound, were employed to confirm cases of mediastinal or intraabdominal masses. Fresh tissue samples were obtained during necropsy, where mediastinal, renal, or intestinal masses were incidentally discovered. Pleural effusion samples were also collected from cats with mediastinal masses, with results indicating lymphoma or strongly suggesting its presence. In cases of mediastinal lymphoma, the inclusion criteria were based on thoracic diagnostic imaging via X-ray, revealing a mass accompanied by pleural effusion.

Eighty-two cases of lymphoma consisted of formalin-fixed and paraffin-embedded biopsy specimens of feline lymphoma (*n* = 49) including extranodal lymphoma (*n* = 18), multicentric lymphoma (*n* = 17), mediastinal lymphoma (*n* = 8), Alimentary lymphoma (*n* = 6). All cell pellets (*n* = 22) obtained from pleural effusion of mediastinal lymphoma. Fresh tissue (*n* = 11) samples collected from multicentric lymphoma (*n* = 5), mediastinal lymphoma (*n* = 4), alimentary lymphoma (*n* = 1) and extranodal lymphoma (*n* = 1). All samples were obtained from different animals. To calculate the sensitivity of PARR in FFPE and fresh tissue groups, 10 true-negative non-lymphoma samples were obtained from cats that were either healthy or had non-lymphoproliferative diseases, including normal lymph nodes (*n* = 4), reactive hyperplastic lymph nodes (*n* = 3), and spleen (*n* = 3). All of them were fresh tissue. For the cell pellet group, 12 true-negative cases were included reactive hyperplastic lymph nodes (*n* = 5), pleural effusion from heart disease (*n* = 4), and pleural effusion in feline infectious diseases confirmed to be caused by feline infectious peritonitis virus using the RT-PCR method (*n* = 3). The control group had a normal hematological profile with no lymphadenopathy. In control cases with pleural effusion, no mediastinal mass was visible on imaging, or cardiovascular signs were observed.

### Cell pellet preparation

Pleural fluid samples were collected via thoracocentesis and placed in ethylenediaminetetraacetic acid (EDTA) tubes to prevent the formation of fibrin clots (22). The samples were then centrifuged at 3000 × g for 5–8 min. The resulting cell pellets were used to prepare four slides, which were air-dried for ICC and Wright-Giemsa staining. Another portion of the cell pellets was stored at-20°C for DNA extraction.

### IHC and ICC

IHC was performed to detect the immunophenotypes of PAX5, CD79α, CD20 (B-cell lineage), and CD3 (T-cell lineage). Forty-nine formalin-fixed, paraffin-embedded lymphoma tissues were prepared on silane-coated slides. For CD3, CD20, and CD79α, the slides were treated with a 1% citrate buffer solution at pH 6.0 in a 95–100°C water bath for 30 min. For PAX5, the slides were treated with a Tris EDTA solution at pH 9 in an autoclave at 121°C for 5 min. Endogenous peroxidase activity was quenched by incubating the sections in 0.3% hydrogen peroxide in phosphate-buffered saline (PBS) for 30 min and blocking them with 2.5% bovine serum albumin at 37°C for 30 min. The sections were then incubated with primary antibodies specific to CD3 (T-cell marker, ready to use, Dako, Denmark), PAX5 (B-cell marker, dilution 1:50, Dako, Denmark), CD79α (B-cell marker, dilution 1:200, SCBT, United States), and CD20 (B-cell marker, dilution 1:300, Abcam, United States) for 90 min, except for PAX5, which was incubated overnight at 4°C. Negative and positive control sections were included at this stage. Normal lymph nodes were used as positive controls, while negative controls had the primary antibody omitted (23). Subsequently, the sections were incubated with EnVision Polymer (Dako, Denmark), and 3,3′-diaminobenzidine was used as a chromogen. The slides were counterstained with Mayer’s hematoxylin. Positive cytoplasmic and/or membranous staining of CD3, CD79α, and CD20 and nuclear staining of PAX5 were evaluated. To diagnose T-cell or B-cell lymphomas, at least 60% of the tumor cells were required to be positive.

ICC was performed on 22 air-dried slides from the pleural effusion of the mediastinal lymphoma. ICC performed by using CD3 for T-cells and CD20 and CD79α for B-cells. The slides were fixed with cold acetone for 2 min, omitting the pre-treatment step. Endogenous peroxidase activity was quenched by incubating the sections in 0.3% hydrogen peroxide for 30 min. The remaining steps followed the same procedure as in IHC, except that the incubation with primary antibodies was performed for 30 min.

### DNA extraction and genomic DNA quality control

Genomic DNA (gDNA) extraction was performed on pleural effusion samples of mediastinal lymphoma using the DNeasy blood and tissue kit (Qiagen, Hilden, Germany). For FFPE samples, the QIAamp DNA FFPE tissue kit (Qiagen, Hilden, Germany) was used according to the manufacturer’s protocol. Pleural effusion samples were centrifuged, and only the fluid was discarded before adding 200 μL of 1 × PBS. For the FNA-derived cytology slides, 200 μL of 1 × PBS and 20 μL of Proteinase K were combined in a 1.5 mL microcentrifuge tube (Eppendorf AG, Hamburg, Germany), and the cell material was scraped off with a sterile blade and transferred quantitatively to the tube. After adding 200 μL lysis buffer (supplied with the kit), the samples were further processed following the kit’s manual. The concentration and quality of the extracted gDNA were assessed using NanoDrop Lite Plus (Thermo Fisher Scientific, Waltham, MA, United States).

For FFPE samples, five pieces of FFPE (each with a size of 10–12 μm) were prepared in a microcentrifuge tube following the manufacturer’s instructions. At least two measurements were performed per gDNA sample to ensure consistency, with the threshold set to 30 ng/μL and desired 260/280 ratios of 1.8–2.0. To assess the suitability of gDNA for the clonality assay, a 550 bp fragment of the feline androgen receptor gene was PCR-amplified for each sample.

All PCR reactions were supplemented with 10 μL of DNA dilution buffer (Qiagen GmbH, Hilden, Germany) and size-separated using the QIAxcel advanced system capillary electrophoresis analyzer with the QIAxcel DNA high-resolution kit and the QX alignment marker 15 bp/1000 bp (Qiagen). The presence and size of the obtained PCR products were accurately determined using QIAxcel ScreenGel software 1.6 (Qiagen).

### Primer and PCR analysis

A reactive volume of 25 μL, including 10 ng of gDNA and a primer mixture as shown in Table 1, 0.65 unit tag primer enzyme (HotStarTag, Qiagen, CA), 2.5x PCR buffer, 5 mM MgCl, 500 mM of each deoxynucleotide triphosphate (dNTP)(Qiagen) and nuclease free water. The cycling conditions of both B-and T-cells were as follows: initial denaturation at 95°C for 15 min, followed by 5 cycles at 94°C for 30 s and 70°C for 60 s, 5 cycles at 94°C for 30 s and 68°C for 60 s, 35 cycles at 94°C for 30 s and 65°C for 60 s, and a final extension at 72°C for 10 min. All PCR reactions were run in triplicate, with positive, negative, and polyclonal control. Both B-cell and T-cell lymphomas served as positive controls. Negative controls omitted DNA, and normal lymph nodes were used as polyclonal controls.

**Table 1:** Primer sets for feline T-cell and B-cell lymphoma targeting TCRG and IGH V genes.

Primer set for TCRG and IGHV gene				Target
T-cell	TCRG V	5′-AAGAGCGAYGAGGGMGTGT-3′	20 pmol	80–120 bp
	TCRG J	5′-CTGAGCAGTGTGCCAGSACC-3′	10 pmol	
B-cell	IGH V	FR2: 5′ -CCAGGCTCCAGGGAAGGG-3′	10 pmol	250–300 bp
	IGH J	J2: 5′-TGAGGACACTGTGAC- TATGGTTCC-3′	10 pmol	
		JD: 5′-GGACACCGTCA-CYAKGVYTCC-3′	100 pmol	
	IGH V	FR3: 5-TCCAGAGACAACGCCAA-GAAC-3′	10 pmol	130–180 bp
	IGH J	J2: 5′-TGAGGACACTGTGAC-TATGGTTCC-3′	10 pmol	
		JD: 5′-GGACACCGTCA-CYAKGVYTCC-3′	100 pmol	

### Clonality

The TCRG rearrangement was evaluated through PCR amplification of the CDR3 located between the V and J segments. In brief, 100 ng of genomic DNA from each sample was subjected to amplification in a 25 mL reaction using a consensus primer derived from the TCRG V segment (5′-AAGAGCGAYGAGGGMGTGT-3′, 20 pmol), along with another primer derived from the TCRG J segment (5′-CTGAGCAGTGTGCCAGSACC-3′, 10 pmol) of the feline TCRG cDNA transcripts (12). Specific amplicons were anticipated to fall within a target range of approximately 80–120 base pairs. The amplification process employed a two-step, modified touchdown protocol to enhance the reactions’ specificity. Each PCR reaction was conducted in triplicate to ensure reliability.

The IGH rearrangement was examined by amplifying the CDR3, which encompassed the V, D, and J segments. In essence, 100 ng of gDNA from each sample was subjected to amplification in a 25 mL reaction vessel using consensus primers derived from the IGH V segment framework 2 (FR2) and framework 3 (FR3) (FR2: 5′-CCAGGCTCCAGGGAAGGG-3′, 10 pmol, and FR3: 5′-TCCAGAGACAACGCCAAAGAAC-3′, 10 pmol), along with consensus primers derived from IGH J segments (J2: 5′-TGAGGACACTGTGACTATGGTTCC-3′, 10 pmol, and JD: 5′-GGACACCGTCACGYAKGVYTCC-3′, 100 pmol). The amplification process employed a two-step, modified touchdown protocol to enhance the specificity of the reactions (13). All samples were analyzed using PCR with both TCRG and IGHV. All PCR reactions were performed in triplicate for consistency.

### Interpretation of clonality pattern

Electropherogram patterns were categorized into four groups: monoclonal, polyclonal, pseudoclonal, and no amplification (11). Monoclonal patterns were characterized by consistent, identical, and sharply defined peaks of the expected size across all three reactions. Polyclonal patterns exhibited a Gaussian curve. Samples showing a distinct, sharp peak within a Gaussian curve, with the dominant clone’s peak height at least twice that of the polyclonal background, were labeled monoclonal in a polyclonal background. Pseudoclonal patterns were identified by peaks of varying sizes, indicating a lack of reproducibility across the three reactions. No amplification was assigned to samples that displayed neither a peak nor a Gaussian curve. For samples labeled clonal in a polyclonal background, it was necessary for at least two out of three (triplicate) results for that sample to show entirely consistent, sharply defined peaks, with each peak being at least twice the height of the polyclonal background in their electrophoresis profiles (14).

### Statistical evaluation

Histopathology and cytology have long served as the gold standards for diagnosing reactive hyperplasia or lymphoma, enabling the identification of true positives, true negatives, false positives, and false negatives in clonality testing results. Using histopathology as the gold standard, true positives, true negatives, false positives, and false negatives were assigned in a 2 × 2 contingency table. Test sensitivity and specificity, including 95% confidence intervals, were calculated (24).

## Results

Most of the samples in this study involved cats under 6 years of age, with domestic short hair being the most commonly represented breed (Table 2 and Supplementary Tables S1–S3). The average gDNA concentration ranged from 12.9 to 1,070.9 ng/μL, with the 260/280 ratios averaging between 1.41 and 1.93. The cell pellets had an average concentration ranging from 59.8 to 501.9 ng/μL. The analysis uncovered diverse clonality patterns within T-cell and B-cell populations, including clonal, bi-clonal, and oligoclonal patterns. To obtain positive results, T-cells should exhibit a narrow peak between 80 and 120 base pairs; this peak can appear once or twice within this range. Three narrow peaks indicate an oligoclonal pattern. Both bi-clonal and oligoclonal patterns are expected to display the same pattern in triplicate samples.

**Table 2:** Comprehensive patient characteristics of feline lymphoma cases.

Patient characteristics		
Age	7 m-3Y	46.3% (38/82)
	4–6 Y	34.2% (28/82)
	> 6Y	19.5% (16/82)
Breed	DSH	96.3% (79/82)
	Persian	2.4% (2/82)
	Scottish Fold	1.3% (1/82)
Sex	Male	47.6% (39/82)
	Female	52.4% (43/82)
Immunophenotype	T-cell lymphoma	35.4% (29/82)
	B-cell lymphoma	64.6% (53/82)

**Figure 1:**
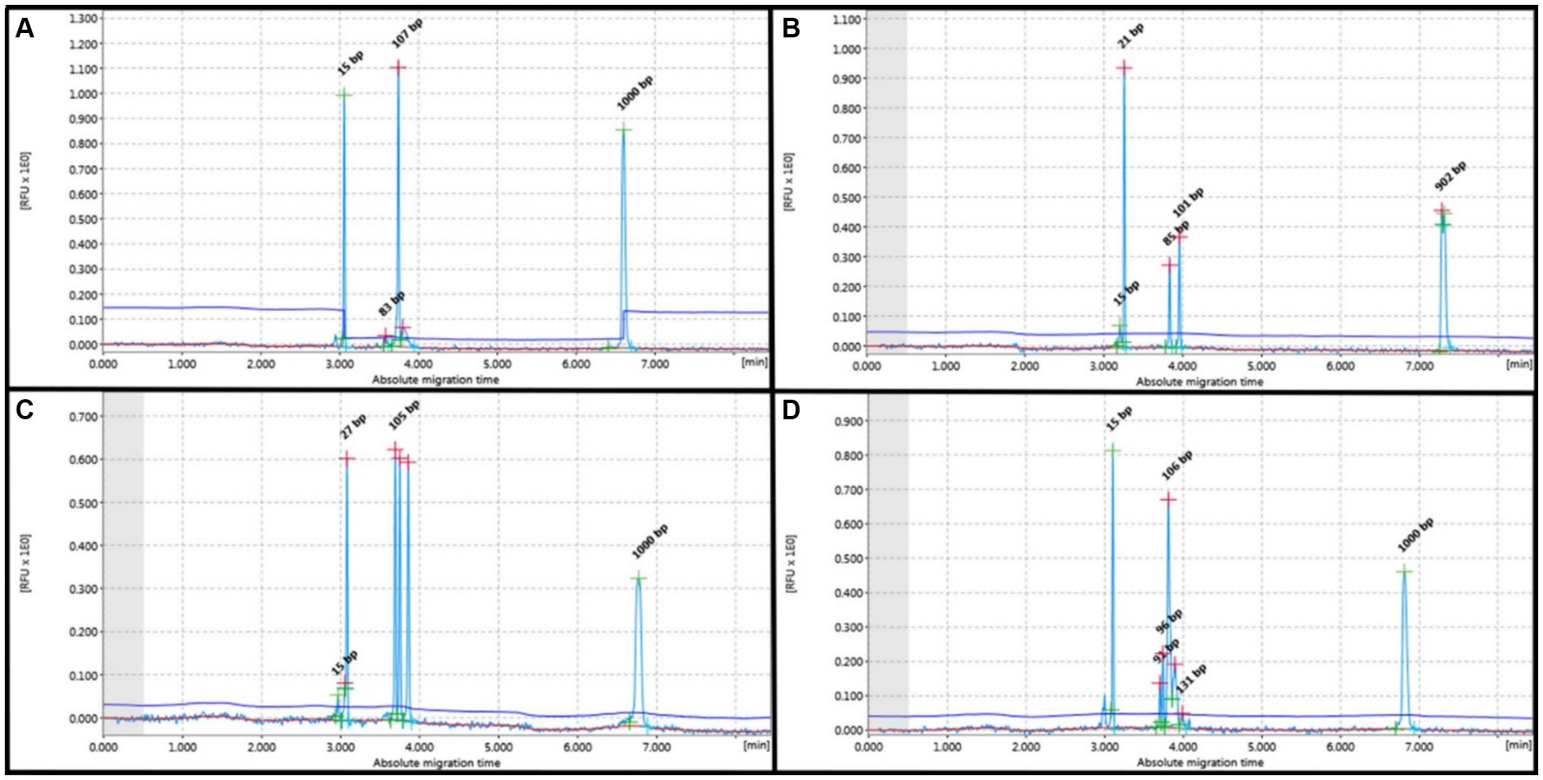
PCR for antigen receptor rearrangement (PARR) using GeneMarker for clonal rearrangement of the T-cell receptor gamma (TCRG). **(A)** Monoclonal displays a narrow peak at 80–120 bp. **(B)** Bi-clonal displays two narrow peaks at 80–120 bp. **(C)** Oligoclonal displays three narrow peaks at 80–120 bp. **(D)** Monoclonal on polyclonal background displays a narrow peak twice the height of the polyclonal background.

**Figure 2:**
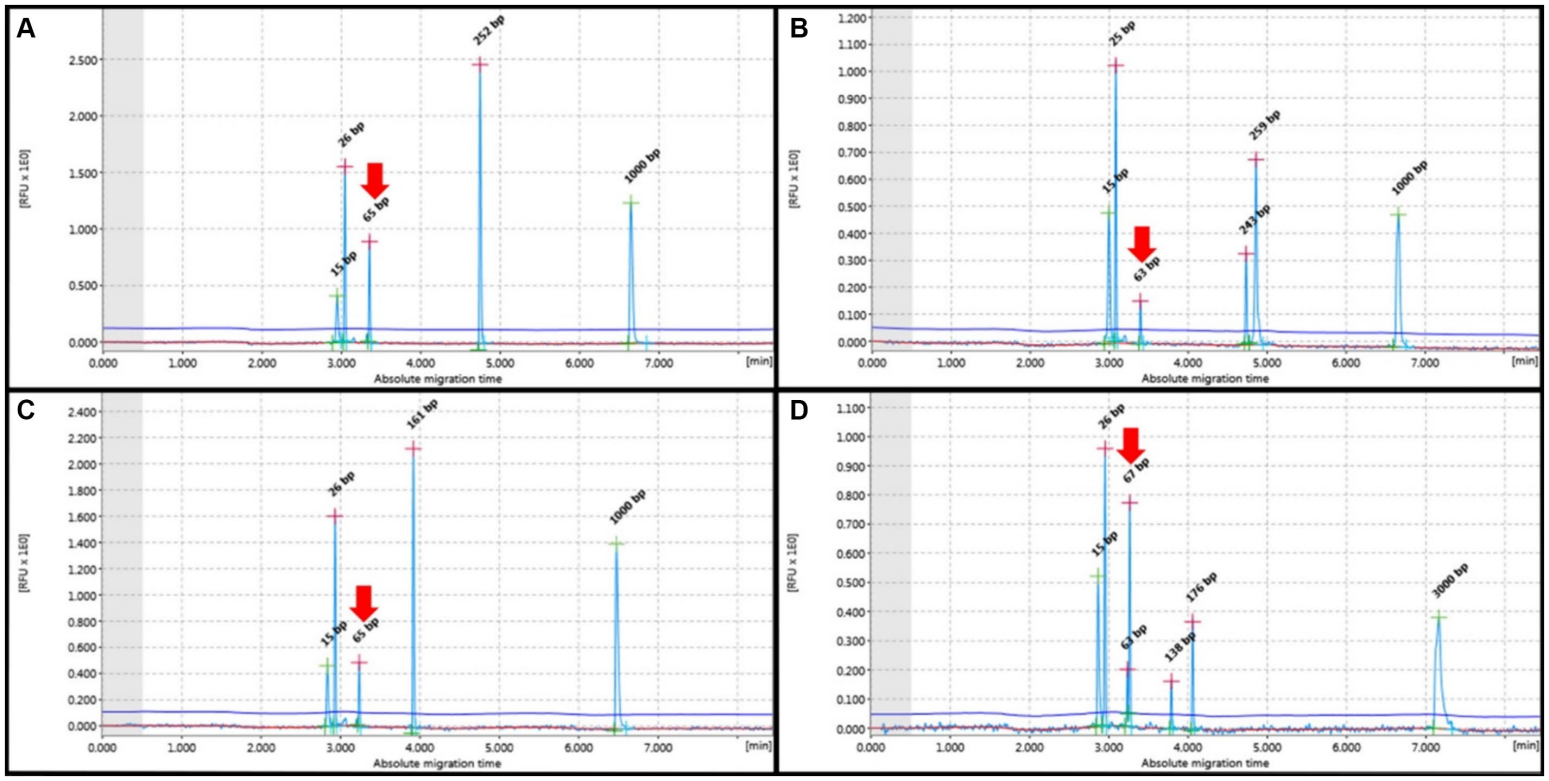
PCR for antigen receptor rearrangement (PARR) GeneMarker for clonal rearrangement form complete immunoglobulin heavy (IGH) chain variable (V)-diversity (D)-joining (J) (IGH-VDJ). **(A)** Monoclonal of the FR2 Primer set showed one narrow peak at 250–300 bp. **(B)** The bi-clonal of the FR2 primer set showed two narrow peaks at 250–300 bp. **(C)** Monoclonal Primer FR3 showed a narrow peak of 130–180 bp. **(D)** Bi-clonal of Primer FR3 displayed two narrow peaks at 130–180 bp. Red arrows indicate non-specific bands not within the target base pair range of the IGH primer set.

The T-cells showed amplification in all cases, and the TCRG pattern demonstrated a monoclonal pattern in 18 out of the 29 cases (62.1%) and a polyclonal pattern in 11 out of the 29 cases (37.9%). Among the monoclonal cases, 83.5% (15/18) were purely monoclonal, 5.5% (1/18) were bi-clonal, 5.5% (1/18) were oligoclonal, and 5.5% (1/18) were clonal in a polyclonal background. For B-cells, we used FR2 and FR3, whose pattern is similar to T-cells but the target base pair ranges differ: FR2 targets 250–300 base pairs, while FR3 targets 130–180 base pairs. In this study, B-cells exhibited only clonal and bi-clonal patterns. Instances of clonality amidst polyclonal backgrounds were also observed. Cases of FR2 showed amplification at 66% (35/53) and no amplification at 34% (18/53); cases of amplification showed a clonal pattern at 60% (21/35) and a polyclonal pattern at 40% (14/35). In addition, 95.2% (20/21) of the IGHV (FR2) showed clonality, while 4.8% (1/21) exhibited a bi-clonal pattern. FR3 showed 75.5% (40/53) amplification and 24.5% (13/53) no amplification. Amplification showed a 55% (22/40) clonal pattern and 45% (16/40) polyclonal pattern. In addition, 95.4% (21/22) of the IGH V (FR3) showed clonality, while 4.5% (1/22) exhibited a bi-clonal pattern. TCRG and IGH results from cell pellets showed a low number (2 out of 22 cases) of no amplification patterns. All data are described in Table 3, and the patterns are shown in Figures 1, 2.

**Table 3:** Conclusion of immunohistochemistry or immunocytochemistry compared with PARR from FFPE, fresh tissue and cell pellets.

Immunohistochemistry/Immunocytochemistry	PCR for Antigen Receptor Rearrangement (PARR)								
	TCRG			IGHV					
				FR2			FR3		
	Clonal	Polyclonal	NA	Clonal	Polyclonal	NA	Clonal	Polyclonal	NA
FFPE (*n* = 49)									
T-cell (*n* = 10)	50% (5/10)	50% (5/10)	0% (0/10)	0% (0/10)	50% (5/10)	50% (5/10)	0% (0/10)	60% (6/10)	40% (4/10)
B-cell (*n* = 39)	0% (0/39)	61.53% (24/39)	38.46% (15/39)	33.3% (13/39)	38.5% (10/39)	28.2% (16/39)	33.33% (13/39)	33.33% (13/39)	33.33% (13/39)
Fresh tissue (*n* = 11)									
T-cell (*n* = 2)	100% (2/2)	0% (0/2)	0% (0/2)	0% (0/2)	100% (2/2)	0% (0/2)	0% (0/2)	100% (2/2)	0% (0/2)
B-cell (*n* = 9)	0% (0/9)	88.9% (8/9)	11.1% (1/9)	66.7% (6/9)	11.1% (1/9)	22.2% (2/9)	88.9% (8/9)	11.1% (1/9)	0% (0/9)
Cell pellets (*n* = 22)									
T-cell (*n* = 17)	64.7% (11/17)	35.3% (6/17)	0% (0/17)	0% (0/17)	88.2% (15/17)	11.8% (2/17)	0% (0/17)	88.2% (15/17)	11.8% (2/17)
B-cell (*n* = 5)	0% (0/5)	100% (5/5)	0% (0/5)	40% (2/5)	60% (3/5)	0% (0/5)	20% (1/5)	80% (4/5)	0% (0/5)
Total									
T-cell (*n* = 29)	62.1% (18/29)	37.9% (11/29)	0% (0/29)	0% (0/29)	75.9% (22/29)	24.1% (7/29)	0% (0/29)	79.3% (23/29)	20.7% (6/29)
B-cell (*n* = 53)	0% (0/53)	69.9% (37/53)	30.1% (16/53)	39.6% (21/53)	26.4% (14/53)	40% (18/53)	41.5% (22/53)	34% (18/53)	24.5% (13/53)

NA, No amplification.

**Figure 3:**
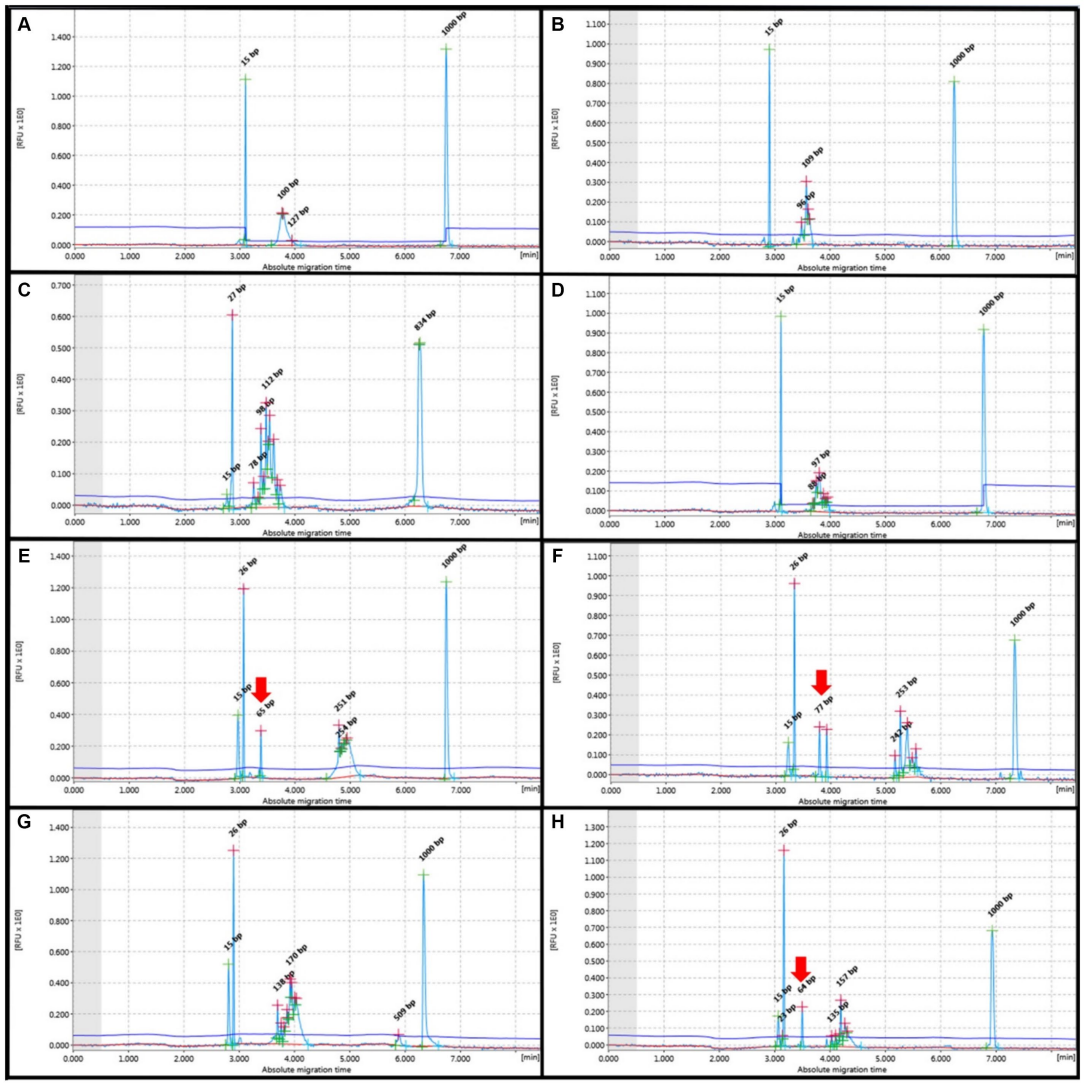
PCR for antigen receptor rearrangement (PARR) GeneMarker for Polyclonal rearrangement form T-cell receptor gamma (TCRG) **(A–D)** showed Gaussian curve without evidence of any non-specific band at 80–120 bp, Complete immunoglobulin heavy (IGH) chain variable (V)-diversity (D)-joining (J) (IGH-VDJ), FR2 **(E,F)** and FR3 **(G,H)** showed Guassian curve at 250–300 bp and 130–180 bp, respectively. Red arrows indicate non-specific bands not within the target base pair range of the IGH primer set.

The polyclonal patterns for both TCRG and IGH displayed Gaussian distribution, including normal Gaussian distribution and multiple peaks in the target range of each primer. Notably, the FFPE group showed the highest frequency of cases with no amplification (19/49). The cell pellet samples displayed Gaussian curves without any instances of no amplification. Non-specific peaks outside the product size range were not observed in TCRG. IGH V exhibited non-specific bands, although they did not fall within the range of the target primer. These bands were observed in all samples, including the positive control. Clonal patterns on polyclonal background where the height did not exceed twice that of the polyclonal background were considered polyclonal (Figure 3).

Comparing IHC and ICC with PARR results in the FFPE samples, the B-cell cases showed concordance in 19 out of 39 instances (48.7%) and discordance in 20 out of 39 cases (51.3%). For T-cell cases, there was concordance in 5 out of 10 instances (50%) and discordance in 5 out of 10 (50%). In fresh tissue samples, B-cell cases showed concordance with IHC in 8 out of 9 instances (88.9%), with only one discordant result (11.1%). All T-cells showed concordance with IHC. In cell pellets, T-cell cases showed concordance in 11 out of 17 instances (64.7%) and discordance in 6 out of 17 (35.3%). For B-cells, there was concordance in 2 out of 5 instances (40%) and discordance in 3 out of 5 instances (60%) (Supplementary Tables S1–S3). Remarkably, fresh tissue samples consistently showed concordant results between the ICC and PCR analyses. However, in some instances, the FFPE samples exhibited no amplification. Furthermore, there was no evidence of cross-lineage rearrangement.

This study compared the sensitivity of IHC and ICC with that of PARR. For TCRG, PARR demonstrated a sensitivity of 58.33% for both fresh tissue and FFPE samples, with a specificity of 100%. Cell pellet analysis exhibited a sensitivity of 64.71% and maintained 100% specificity. A combined analysis of fresh tissue, FFPE, and cell pellets showed a sensitivity of 62.07%. For IGH, the sensitivity for both fresh tissue and FFPE samples was 56.25%, while cell pellet analysis showed a sensitivity of 62.50%. However, when considering fresh tissue and FFPE with cell pellet samples, the sensitivity was 57.14%. The sensitivity data are described in detail in Tables 4, 5.

**Table 4:** Results of clonality testing for TCRG using different sample specimens.

TCRG	Fresh tissue and FFPE		Cell pellets		Fresh tissue, FFPE, and cell pellets	
Statistic	Value (%)	95% CI	Value (%)	95% CI	Value (%)	95% CI
Sensitivity	58.3	27.67–84.83	64.71	38.33–85.79	62.07	42.26–79.31
Specificity	100.00	73.54–100.00	100.00	73.54–100.00	100.00	84.56–100.00
PPV	100.00	59.04–100.00	100.00	71.51–100.00	100.00	81.47–100.00
NPV	70.59	55.13–82.42	66.67	51.24–79.20	66.67	55.66–76,11

CI, Confidence interval; PPV, Positive predictive value; NPV, Negative predictive value.

**Table 5:** Results of clonality testing for IGH V using different sample specimens.

IGH	Fresh tissue and FFPE		Cell pellets		Fresh tissue, FFPE, and cell pellets	
Statistic	Value (%)	95% CI	Value (%)	95% CI	Value (%)	95% CI
Sensitivity	56.25	41.18–70.52	62.50	24.49–91.48	57.14	43.22–70.29
Specificity	100.00	69.15–100.00	100.00	73.54–100.00	100.00	84.56–100.00
PPV	100.00	87.23–100.00	100.00	47.82–100.00	100.00	89.11–100.00
NPV	32.26	26.68–39.62	80.00	62.11–96.79	47.83	40.39–55.36

CI, Confidence interval; PPV, Positive predictive value; NPV, Negative predictive value.

Two cases included samples from all three groups: cell pellets derived from pleural effusion, fresh tissue from the necropsy room, and FFPE. All samples from both cases were from mediastinal lymphoma and showed consistent T-cell results with both IHC and PARR.

**Figure 4:**
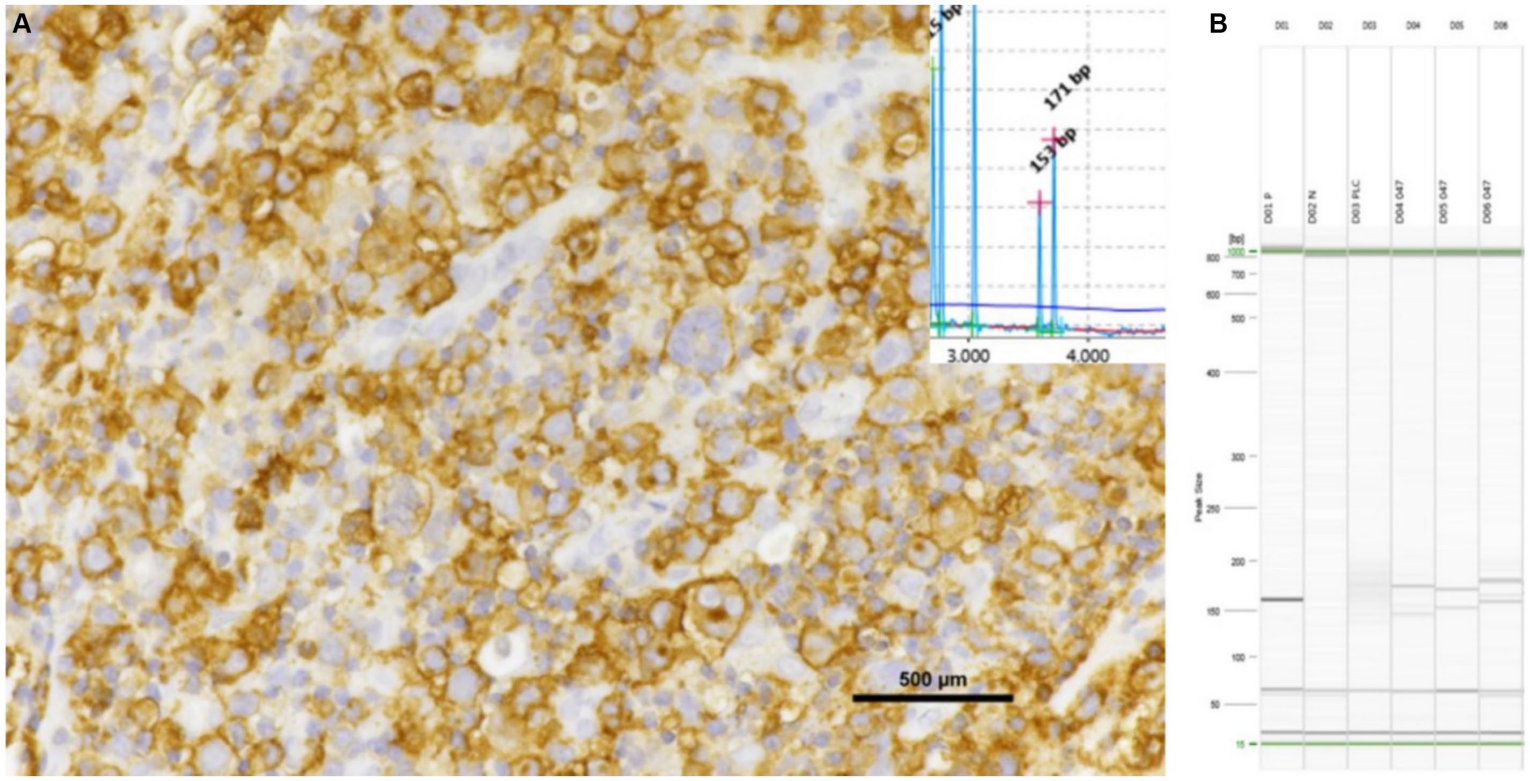
Inguinal lymph node with positive for CD20 shows the results of PCR assays. The samples exhibited a bi-clonal pattern on both QIAxcel **(A)** and gel images **(B)**, utilizing the FR3 primer set of the IGH V. All triplicate samples exhibited the same band at 130–180 bp.

**Figure 5:**
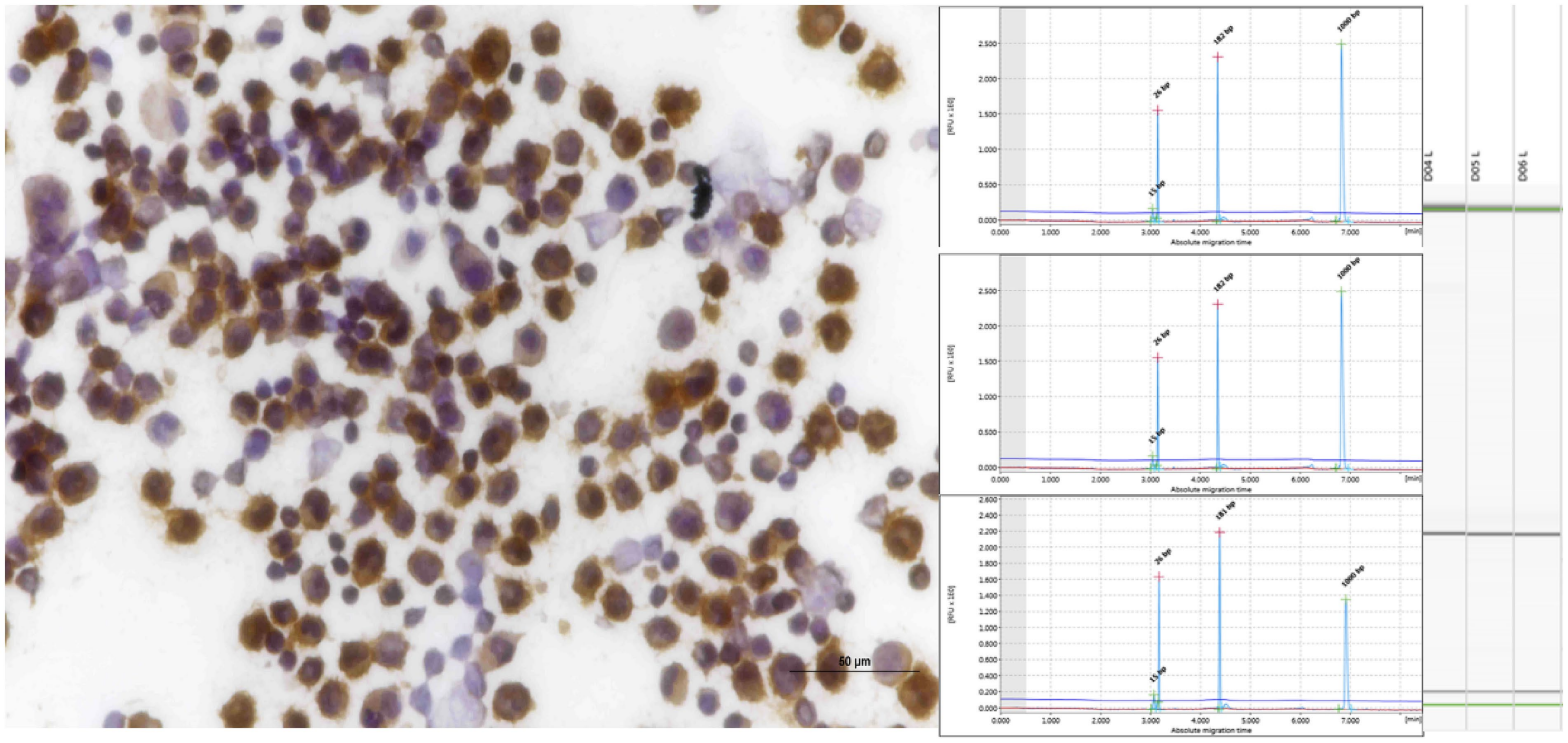
Pleural effusion from mediastinal lymphoma samples that tested positive for CD20 exhibited clonality when analyzed using IGH (FR3), as evidenced by both QIAxcel and gel images.

**Figure 6:**
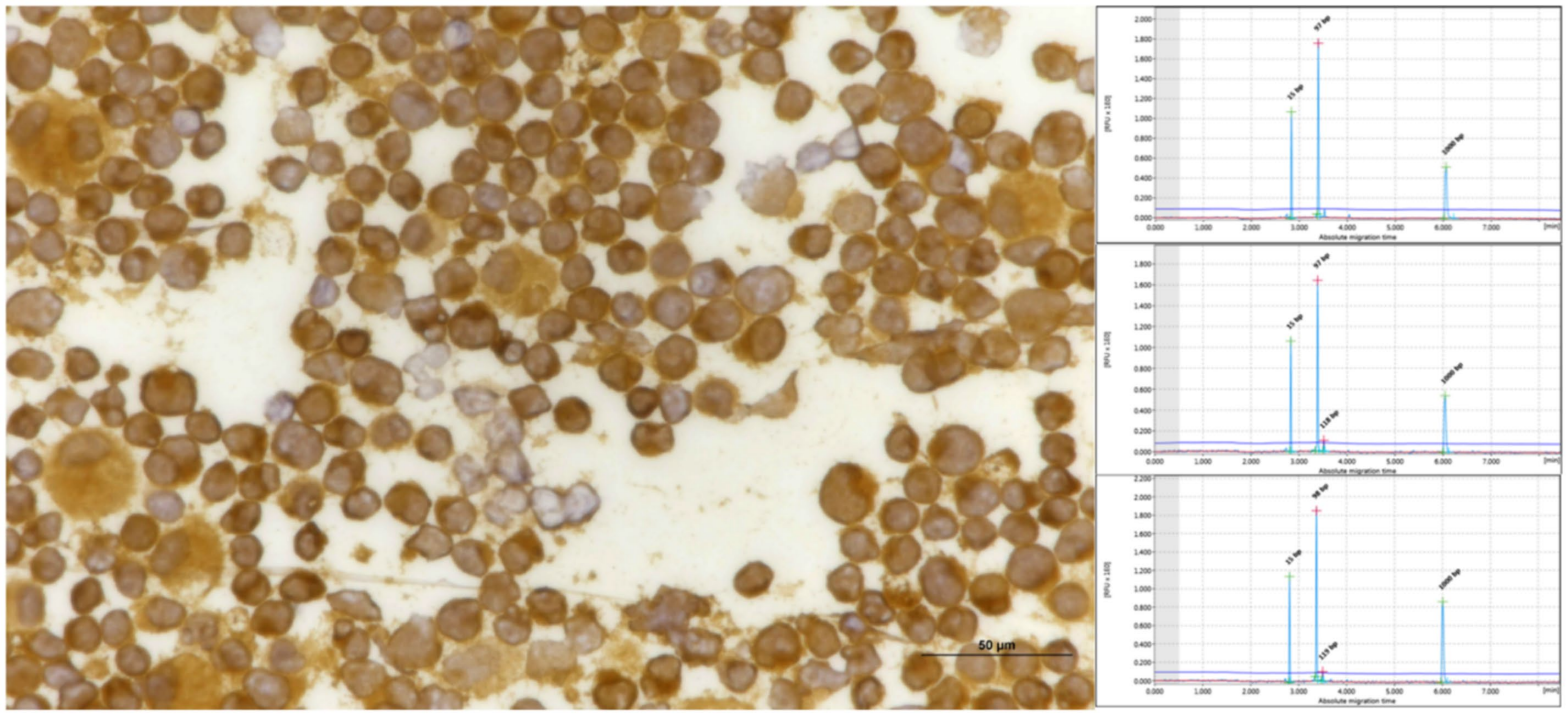
Pleural effusion from mediastinal lymphoma samples that tested positive for CD3 exhibited triplicate clonality when analyzed using TCRG, as evidenced by QIAxcel.

Notably, clonality for both TCRG and IGH V was detected in cell pellets from the pleural effusion of mediastinal lymphoma cases, although fresh tissue was deemed to provide more conclusive results. FNA from a mass in the pleural space yielded more specific results than pleural effusion. Fresh tissue, FFPE, and cell pellets from pleural effusion are all potential specimens for PARR analysis (Figures 4–6). However, each sample type presents specific issues, which will be discussed later.

## Discussion

Clonality could be detected in approximately 34–89% of feline B-cell neoplasms (2). Conversely, PCR-based clonality assays targeting clonal TCRG gene rearrangements demonstrated a sensitivity of up to 90%, particularly in intestinal forms (5, 10, 15). However, false negative and positive results may occur (10). This study found that the sensitivity was 62.07% for T-cells and 57.14% for B-cell neoplasms, consistent with the sensitivity range observed in previous studies (2, 10, 15, 16). Although this study showed a lower sensitivity for T-cell neoplasms, it still fell within the expected range. Furthermore, the samples in this study encompassed various anatomical classifications. The differences in sensitivity compared to previous studies were attributable to variations in primer selection, sample types, and experimental conditions.

FFPE samples often exhibit numerous non-amplifications attributable to paraffin defects and DNA damage. However, GAPDH consistently manifests good sample quality. In this study, in cases where both fresh and FFPE samples were available, there were notable differences in the outcomes of both extraction and PARR runs. While fresh tissue samples yielded precise results, FFPE samples showed no amplification.

In this study, we set a threshold for the gDNA concentration at 30 ng/μL. However, lower gDNA concentrations, such as 12.9 ng/μL, also yielded good results in some cases. Previous studies have indicated that the extracted gDNA should have a minimum concentration of 50 ng/μL with 260/280 ratios between 1.8 and 2.0 (5). Another study established a 30 ng/μL threshold with desired 260/280 ratios of 1.8–2.0 (1).

A few studies have demonstrated clonality patterns from cell pellets of pleural effusion of mediastinal lymphoma with favorable results; however, they lacked additional details (2, 21). This study collected samples from both pleural effusion and mediastinal mass, showing consistent findings. Collecting pleural effusion samples via thoracocentesis from mediastinal masses was easier than directly sampling the masses. The results were generally good for TCRG, but IGH sometimes yielded inconclusive results.

Based on the cell pellet samples, good sensitivity was observed in both T-cell and B-cell groups. However, in the T-cell group positive for CD3, some cases exhibited polyclonal results with both primers. Therefore, an alternative primer is required for re-evaluation in this subgroup. Another primer for TCRG has been developed to detect clonal TCR gene rearrangement in feline lymphoid neoplasms, providing a valuable, high-fidelity molecular diagnostic tool for feline T-cell neoplasms (15). TCRG 2 has been designed to aid in detecting T-cell lymphoma, showing improved performance over TCRG 1 (14). Additionally, alternative primer sets have been used for B-cell lymphoma to enhance sensitivity (1, 2, 16). The new primer sets for B-and T-cells should be assessed for this sample group to determine the most suitable one for obtaining precise results.

The pleural effusion of mediastinal lymphoma demonstrated good sensitivity, especially in cases involving T-cell analysis, where, despite small sample sizes, all results were exact. However, some cases showed differences, indicating that both the quantity and quality of the sample are crucial for obtaining reliable results.

Additionally, when compared with the FFPE samples, the FFPE cases in this study may have experienced DNA damage due to formalin fixation. Gress et al. outlined the criteria for good quality FFPE samples and that the QIAamp DNA FFPE tissue kit (Qiagen, Hilden, Germany) includes heating at 90°C for 60 min to resolve crosslinking among nucleic acids, proteins, and formalin (5). However, some cases in this study still showed no amplification compared to fresh tissue, perhaps due to formalin fixation exceeding 48 h.

Nasal lymphoma positive for B-cells exhibited clonality for FR3, but for FR2, the majority showed polyclonal results, with no amplification. However, due to the limited number of nasal lymphoma cases studied, it cannot be concluded that nasal lymphomas are predominantly sensitive to the FR3 primer set. This aspect has not been addressed in previous studies.

In Thailand, there has been a notable increase in feline leukemia virus (FeLV) infection (25). FeLV, a gammaretrovirus, is widely recognized as the leading cause of feline lymphoma (26). In cases of mediastinal lymphoma, it is often attributed to both a lack of vaccination and breed predispositions. FeLV data were lacking in this study, as this information including FeLV and FIV status and vaccination history was not provided.

In conclusion, molecular techniques have emerged as valuable tools for detecting lymphoma, especially in cases where traditional diagnostic methods yield inconclusive results (9, 18). While biopsy may not always be feasible, cytology and cell pellets obtained from pleural effusion offer alternative ICC and molecular analysis samples, assuming sufficient quality and quantity. This study indicates that all sample types are suitable for PARR to aid in cases with inconclusive results. However, each sample type presents its limitations, as outlined earlier. Therefore, the sample selection should be tailored to the clinical situation.

## Data Availability

The original contributions presented in the study are included in the article/Supplementary material, further inquiries can be directed to the corresponding author.
