# Supplementary material for: Investigating comparative polymerase chain reaction for antigen receptor rearrangement analysis in different types of feline lymphoma samples
Source: Front Vet Sci. 2024 Aug 30;11:1439068. doi: 10.3389/fvets.2024.1439068 (PMC11392920; doi:10.3389/fvets.2024.1439068)
Supplement: Supplementary file 1 [file Table_1.DOCX]

Supplement

Table 1 Patient characteristics, immunophenotype, and clonality results of feline lymphoma from FFPE samples

| No. | Breed | Sex | Age (Y) | Anatomical location | Immunophenotype | Clonality | | |
| --- | --- | --- | --- | --- | --- | --- | --- | --- |
|  |  |  |  |  | IHC | TCRG | IGH(FR2) | IGH(FR3) |
| 1 | DSH | M | 1 | Multicentric | B-cell | NA | Monoclonal | Monoclonal |
| 2 | DSH | F | 5 | Multicentric | B-cell | NA | Monoclonal | Monoclonal |
| 3 | DSH | M | 4 | Mediastinal | B-cell | NA | NA | NA |
| 4 | DSH | M | 1 | Multicentric | B-cell | NA | NA | NA |
| 5 | DSH | M | 1 | Multicentric | B-cell | NA | Monoclonal | Monoclonal |
| 6 | DSH | M | 2 | Multicentric | B-cell | NA | Monoclonal | Monoclonal |
| 8 | DSH | F | 6 | Multicentric | B-cell | NA | NA | Monoclonal |
| 10 | DSH | F | 4 | Multicentric | B-cell | NA | Monoclonal | Polyclonal |
| 11 | DSH | M | 5 | Multicentric | B-cell | Polyclonal | Monoclonal | Polyclonal |
| 15 | DSH | M | 4 | Multicentric | B-cell | Polyclonal | Monoclonal | Monoclonal |
| 16 | DSH | F | 1 | Alimentary | T-cell | Polyclonal | Polyclonal | Polyclonal |
| 25 | DSH | M | 10 | Alimentary | T-cell | Polyclonal | NA | NA |
| 26 | DSH | M | 18 | Multicentric | T-cell | Polyclonal | Polyclonal | Polyclonal |
| 30 | DSH | M | 2 | Multicentric | B-cell | NA | Polyclonal | Polyclonal |
| 32 | DSH | M | 7 | Multicentric | B-cell | NA | Polyclonal | Polyclonal |
| 36 | DSH | F | 1 | Mediastinal | T-cell | Monoclonal | Polyclonal | Polyclonal |
| 37 | DSH | M | 8 | Extranodal (Ocular) | B-cell | Polyclonal | Monoclonal | Monoclonal |
| 38 | DSH | M | 1 | Mediastinal | T-cell | Polyclonal | NA | Polyclonal |
| 41 | PS | M | 2 | Extranodal(Skin) | B-cell | Polyclonal | Polyclonal | Polyclonal |
| 42 | DSH | F | 2 | Multicentric | T-cell | Monoclonal | Polyclonal | Polyclonal |
| 43 | DSH | F | 1 | Mediastinal | T-cell | Monoclonal | NA | NA |
| 44 | DSH | F | 5 | Extranodal (Ocular) | B-cell | NA | NA | NA |
| 45 | DSH | F | 4 | Extranodal (Skin) | B-cell | NA | NA | Polyclonal |
| 46 | DSH | F | 2 | Extranodal (Skin) | B-cell | Polyclonal | NA | NA |
| 47 | DSH | M | 2 | Extranodal (Ocular) | T-cell | Polyclonal | NA | NA |
| 49 | DSH | F | 7 | Extranodal (Skin) | B-cell | NA | NA | NA |
| 50 | DSH | F | 8 | Extranodal (Kidney) | B-cell | Polyclonal | Monoclonal | Monoclonal |
| 52 | DSH | F | 4 | Mediastinal | B-cell | NA | NA | NA |
| 53 | DSH | M | 4 | Alimentary | B-cell | NA | NA | NA |
| 54 | DSH | M | 5 | Extranodal (Nasal) | B-cell | Polyclonal | Polyclonal | Monoclonal |
| 56 | DSH | F | 5 | Extranodal (Nasal) | B-cell | Polyclonal | Polyclonal | Monoclonal |
| 58 | DSH | M | 1 | Mediastinal | T-cell | Monoclonal | Polyclonal | Polyclonal |
| 59 | DSH | M | 1 | Multicentric | B-cell | Polyclonal | Polyclonal | Polyclonal |
| 60 | DSH | M | 1 | Mediastinal | B-cell | Polyclonal | Monoclonal | Polyclonal |
| 62 | DSH | F | 13 | Alimentary | B-cell | Polyclonal | Polyclonal | Polyclonal |
| 68 | DSH | Mc | 11 | Multicentric | B-cell | Polyclonal | NA | NA |
| 69 | DSH | F | 2 | Mediastinal | B-cell | Polyclonal | Polyclonal | Polyclonal |
| 70 | DSH | F | 3 | Extranodal (Nasal) | B-cell | Polyclonal | NA | NA |
| 71 | DSH | F | 6 | Extranodal (Nasal) | B-cell | Polyclonal | Polyclonal | Monoclonal |
| 74 | DSH | Mc | 9 | Alimentary | B-cell | Polyclonal | Monoclonal | Polyclonal |
| 76 | DSH | Fs | 5 | Extranodal (Nasal) | B-cell | Polyclonal | Polyclonal | Polyclonal |
| 78 | SF | F | 1 | Extranodal (Kidney) | B-cell | Polyclonal | NA | NA |
| 79 | DSH | Mc | 11 | Extranodal (Skin) | B-cell | Polyclonal | Monoclonal | NA |
| 80 | DSH | F | 9 | Multicentric | B-cell | Polyclonal | Monoclonal | NA |
| 82 | DSH | M | 4 | Extranodal (Nasal) | B-cell | Polyclonal | NA | Monoclonal |
| 83 | DSH | F | 4 | Extranodal (Nasal) | B-cell | Polyclonal | NA | Monoclonal |
| 84 | DSH | F | 5 | Multicentric | T-cell | Monoclonal | NA | NA |
| 85 | DSH | M | 3 | Alimentary | B-cell | Polyclonal | NA | NA |
| 87 | DSH | M | 4 | Extranodal (Nasal) | B-cell | Polyclonal | NA | Polyclonal |

- DSH= domestic short hair, M= Male, Mc= male castrated, F=Female, Fs= female sprayed, Y=years, NA= No amplification

Table 2 Patient characteristics, immunophenotype, and clonality results of feline lymphoma from fresh tissue samples

| No. | Breed | Sex | Age(Y) | Anatomical location | Immunophenotype | Clonality | | |
| --- | --- | --- | --- | --- | --- | --- | --- | --- |
|  |  |  |  |  | IHC | TCRG | IGH(FR2) | IGH(FR3) |
| 12 | DSH | Fs | 4 | Multicentric | B-cell | Polyclonal | Monoclonal | Monoclonal |
| 13 | DSH | F | 3 | Multientric | B-cell | Polyclonal | Monoclonal | Monoclonal |
| 14 | DSH | F | 10 | Mediastinal | T-cell | Monoclonal | Polyclonal | Polyclonal |
| 48 | DSH | F | 8 | Mediastinal | B-cell | NA | Monoclonal | Monoclonal |
| 61 | DSH | Fs | 5 | Alimentary | B-cell | Polyclonal | Monoclonal | Monoclonal |
| 63 | DSH | F | 1 | Multicentric | B-cell | Polyclonal | NA | Monoclonal |
| 64 | DSH | M | 4 | Mediastinal | T-cell | Monoclonal | Polyclonal | Polyclonal |
| 65 | DSH | F | 6 | Multicentric | B-cell | Polyclonal | Polyclonal | Polyclonal |
| 66 | DSH | Fs | 3 | Multientric | B-cell | Polyclonal | NA | Monoclonal |
| 67 | DSH | Fs | 8 | Mediastinal | B-cell | Polyclonal | Monoclonal | Monoclonal |
| 72 | DSH | Fs | 8 | Extranodal (Kidney) | B-cell | Polyclonal | Monoclonal | Monoclonal |

- DSH= domestic short hair, M= Male, Mc= male castrated, F=Female, Fs= female sprayed, Y=years, NA= No amplification

Table 3 Patient characteristics, immunophenotype, and clonality results of feline lymphoma from cell pellet samples.

| No. | Breed | Sex | Age (y) | Sample Type | Immunophenotype | Clonality | | |
| --- | --- | --- | --- | --- | --- | --- | --- | --- |
|  |  |  |  |  | ICC | TCRG | IGH(FR2) | IGH(FR3) |
| 89 | DSH | M | 3 | PF | T-cell | Polyclonal | Polyclonal | Polyclonal |
| 90 | DSH | M | 3 | PF | T-cell | Polyclonal | Polyclonal | Polyclonal |
| 91 | DSH | M | 5 | PF | B-cell | Polyclonal | Monoclonal | Monoclonal |
| 92 | DSH | M | 5 | PF | T-cell | Polyclonal | Polyclonal | Polyclonal |
| 93 | DSH | M | 2 | PF | T-cell | Monoclonal | NA | NA |
| 94 | DSH | F | 3 | PF | B-cell | Polyclonal | Polyclonal | Polyclonal |
| 95 | DSH | F | 3 | PF | T-cell | Monoclonal | NA | NA |
| 103 | PS | M | 6 | PF | T-cell | Polyclonal | Polyclonal | Polyclonal |
| 104 | DSH | M | 7m | PF | T-cell | Monoclonal | Polyclonal | Polyclonal |
| 105 | DSH | M | 4 | PF | T-cell | Monoclonal | Polyclonal | Polyclonal |
| 106 | DSH | F | 4 | PF | B-cell | Polyclonal | Polyclonal | Polyclonal |
| 107 | DSH | F | 4 | PF | T-cell | Monoclonal | Polyclonal | Polyclonal |
| 108 | DSH | M | 1 | PF | T-cell | Monoclonal | Polyclonal | Polyclonal |
| 109 | DSH | M | 1 | PF | T-cell | Monoclonal | Polyclonal | Polyclonal |
| 110 | DSH | F | 1 | PF | T-cell | Monoclonal | Polyclonal | Polyclonal |
| 111 | DSH | F | 3 | PF | T-cell | Monoclonal | Polyclonal | Polyclonal |
| 112 | DSH | F | 3 | PF | T-cell | Polyclonal | Polyclonal | Polyclonal |
| 113 | DSH | F | 10 | PF | B-cell | Polyclonal | Polyclonal | Polyclonal |
| 114 | DSH | M | 3 | PF | B-cell | Polyclonal | Monoclonal | Polyclonal |
| 115 | DSH | M | 3 | PF | T-cell | Polyclonal | Polyclonal | Polyclonal |
| 116 | DSH | F | 3 | PF | T-cell | Monoclonal | Polyclonal | Polyclonal |
| 117 | DSH | F | 1 | PF | T-cell | Monoclonal | Polyclonal | Polyclonal |

- DSH= domestic short hair, M= Male, Mc= male castrated, F=Female, Fs= female sprayed, Y=years, PF= Pleural effusion , BF= Buffy coat smear ,NA= No amplification
