# Supplementary material for: Investigating comparative polymerase chain reaction for antigen receptor rearrangement analysis in different types of feline lymphoma samples
Source: Front Vet Sci. 2024 Aug 30;11:1439068. doi: 10.3389/fvets.2024.1439068 (PMC11392920; doi:10.3389/fvets.2024.1439068)
Supplement: Supplementary file 2 [file Table_2.DOCX]

Supplementary


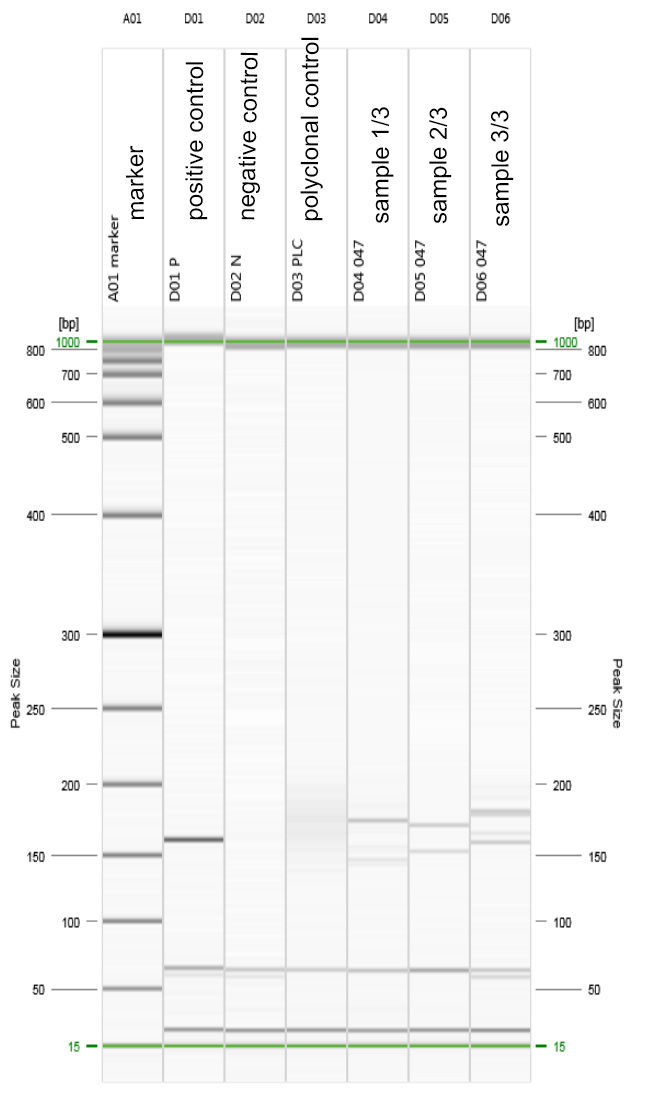


The whole gel image shown in Figure 4 consists of, from left to right: a marker, a positive control, a negative control, a polyclonal control, and a triplicate sample with biclonal results. The gel image was generated using QIAxcel ScreenGel Software 1.6 (Qiagen).


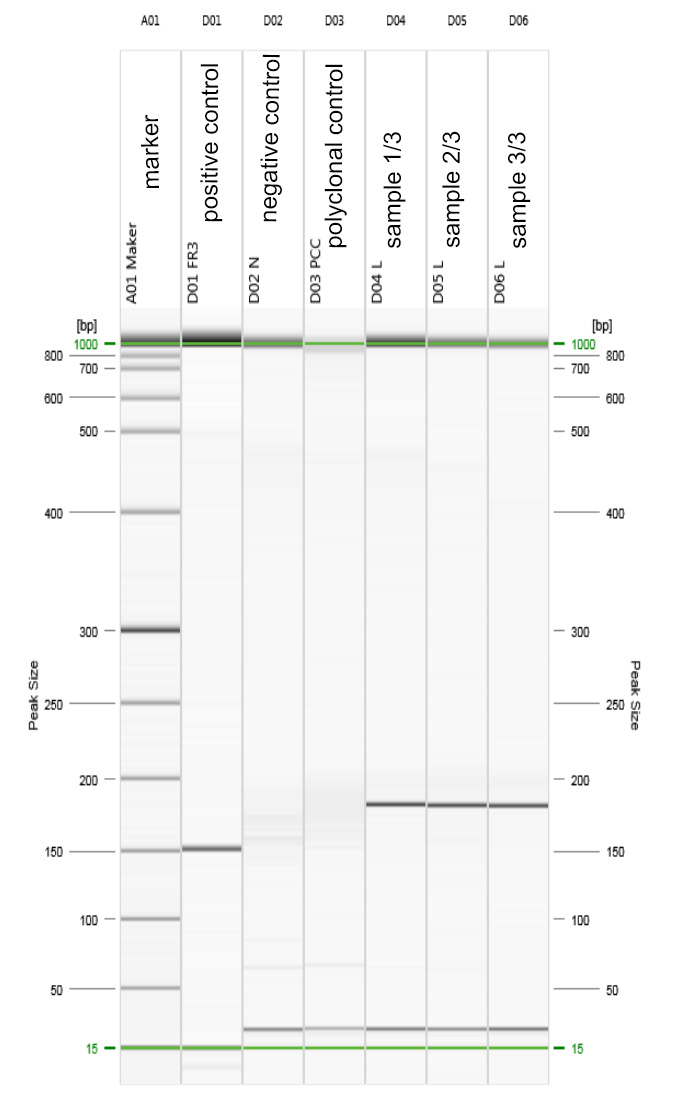


The whole gel image shown in Figure 5 consists of, from left to right: a marker, a positive control, a negative control, a polyclonal control, and a triplicate sample with monoclonal results. The gel image was generated using QIAxcel ScreenGel Software 1.6 (Qiagen).


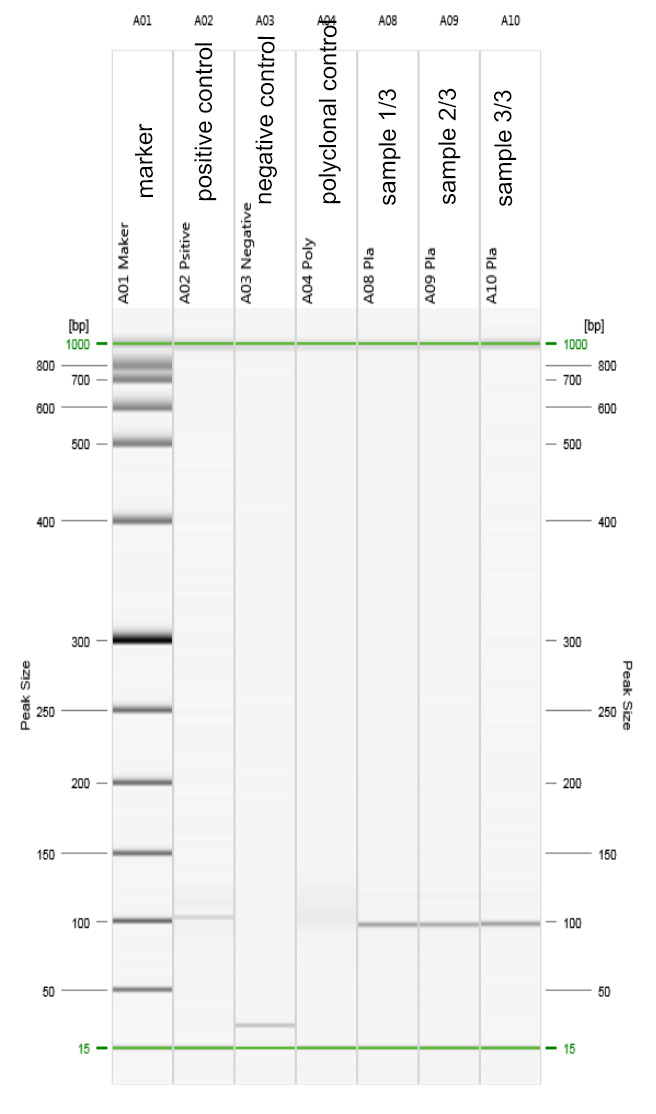


The whole gel image of Figure 6 consists of, from left to right: a marker, a positive control, a negative control, a polyclonal control, and a triplicate sample with monoclonal results. The gel image was generated using QIAxcel ScreenGel Software 1.6 (Qiagen).
