# Supplementary figures and images for: Investigating comparative polymerase chain reaction for antigen receptor rearrangement analysis in different types of feline lymphoma samples
Source: Front Vet Sci. 2024 Aug 30;11:1439068. doi: 10.3389/fvets.2024.1439068 (PMC11392920; doi:10.3389/fvets.2024.1439068)

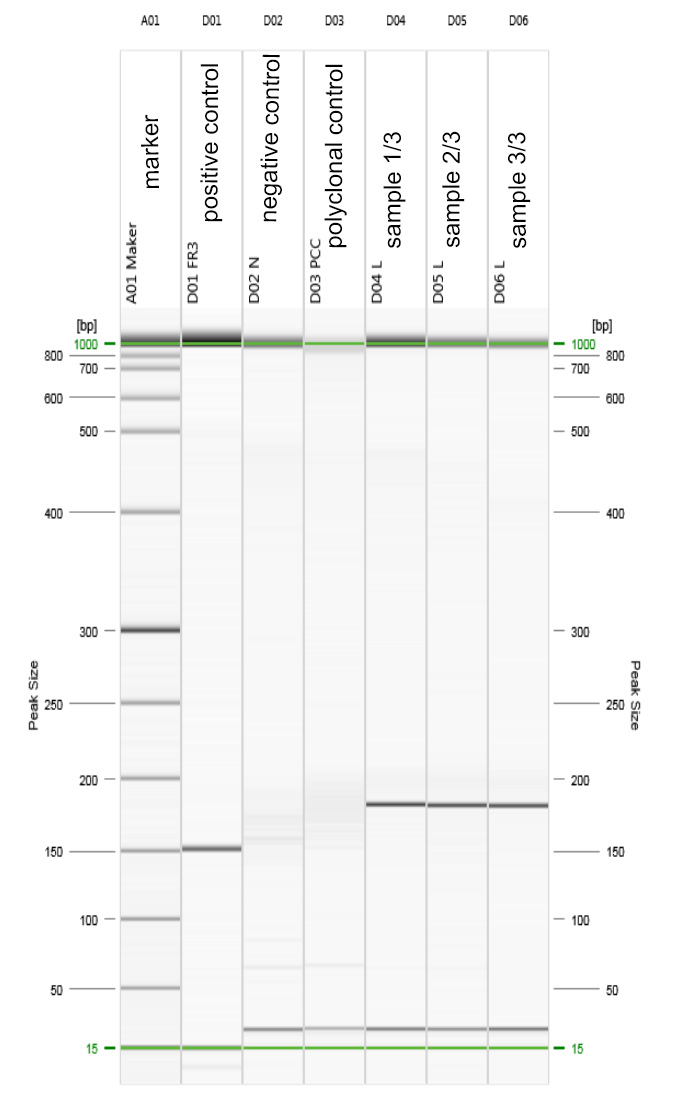

Supplement: Supplementary file 3 [file Image_1.JPEG]

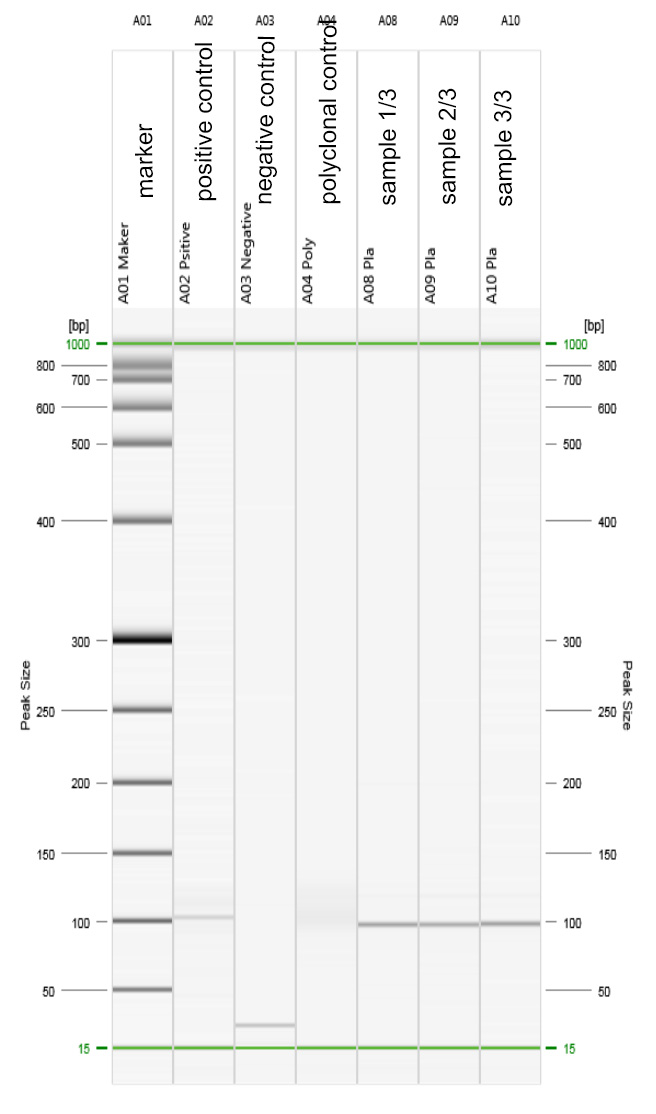

Supplement: Supplementary file 4 [file Image_2.JPEG]

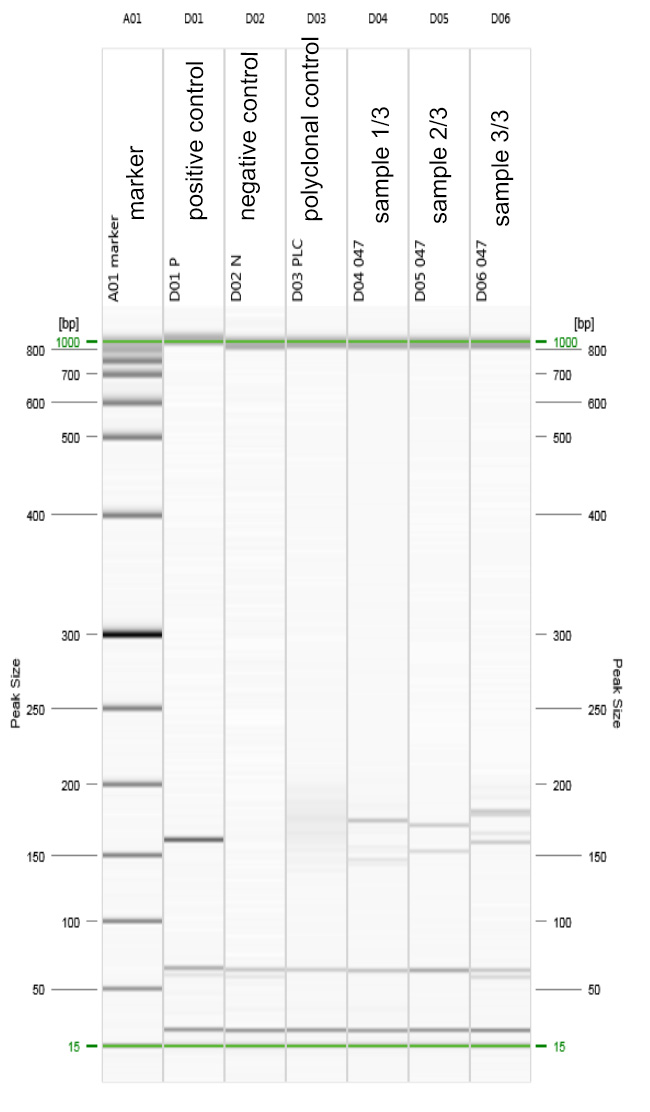

Supplement: Supplementary file 5 [file Image_3.JPEG]
